# Roles of CDR2 and CDR2L in Anti-Yo Paraneoplastic Cerebellar Degeneration: A Literature Review

**DOI:** 10.3390/ijms26010070

**Published:** 2024-12-25

**Authors:** Pablo S. Martínez Lozada, Rafael Mancero Montalvo, Andrea Iturralde Carrillo, Maria Montesdeoca-Lozada, Jose A. Rodas, Jose E. Leon-Rojas

**Affiliations:** 1 NeurALL Research Group, Quito 170157, Ecuador; martinez.sebastian2503@gmail.com; 2 Medical School, Universidad Internacional del Ecuador (UIDE), Quito 170411, Ecuador; 3 School of Psychology, University College Dublin, D04 V1W8 Dublin, Ireland; 4 Escuela de Psicología, Universidad Espíritu Santo, Samborondón 092301, Ecuador; 5 Cerebro, Emoción y Conducta, School of Medicine, Universidad de las Américas (UDLA), Quito 170124, Ecuador

**Keywords:** paraneoplastic cerebellar degeneration, anti-Yo, neuroimmunology, CDR2, CDR2L

## Abstract

Paraneoplastic cerebellar degeneration (PCD) is a rapidly progressive, immune-mediated syndrome characterized by the degeneration of Purkinje cells, often associated with the presence of antibodies targeting intracellular antigens within these cells. These autoantibodies are implicated in the induction of cytotoxicity, leading to Purkinje cell death, as demonstrated in in vitro models. However, the precise roles of antibodies and T lymphocytes in mediating neuronal injury remain a subject of ongoing research, with T cells appearing to be the main effectors of cerebellar injury. Notably, at least 50% of PCD cases involve anti-Yo autoantibodies, also referred to as anti-PCA1 (Purkinje cell antigen 1) antibodies, which specifically target cerebellar degeneration-related protein 2 (CDR2) and its paralogue, CDR2-like (CDR2L). Another recognized antigen is CDR 34, a 34 kDa Purkinje cell antigen characterized by tandem repeats and a B-cell epitope; its detection in non-cerebellar tissues necessitates further in situ hybridization studies. Onconeural antigens are expressed in both Purkinje cells and tumour cells, where they localize in the cytoplasm and associate with membrane-bound and free ribosomes, playing critical roles in regulating transcription and calcium homeostasis. Recent studies suggest that the breakdown of immune tolerance is linked to genetic alterations in tumour cell antigens, leading to the formation of neoantigens that can elicit autoreactive T cells, which may underscore the function of Yo antibodies. In vitro studies indicate that anti-Yo antibodies can induce cell death independent of T lymphocytes. The disease progresses by initial lymphocytic infiltration, followed by a rapid loss of Purkinje cells without significant inflammation. However, in vivo models showcase that anti-Yo PCD is primarily T-cell mediated, with antibodies serving as biomarkers rather than direct effectors of neuronal death. This review examines the mechanisms underlying PCD, focusing on the roles of CDR2 and CDR2L in tumour development and their potential role in the degeneration of cerebellar Purkinje neurons. A comprehensive understanding of these processes is essential for advancing diagnostic, prognostic, and therapeutic strategies for PCD and associated malignancies.

## 1. Introduction

Anti-Yo paraneoplastic cerebellar degeneration (PCD) is a subacute ataxia characterized by the selective degeneration of Purkinje cells in the cerebellum, resulting from immune-mediated cross-reactions. In the cerebellum, Purkinje cells are large, GABAergic neurons with extensive dendritic arborizations, receiving excitatory input from granule cells and climbing fibres and sending inhibitory signals to the deep cerebellar nuclei, playing a crucial role in motor coordination and balance regulation; this degeneration of Purkinje cells contributes to the hallmark presentation of cerebellar ataxia [1]. Anti-Yo PCD is a pan-cerebellar syndrome, presenting with trunk and limb ataxia and often accompanied by brainstem symptoms; clinical stabilization typically occurs within six months [1,2,3]. Cognitive and psychiatric impairments are common in affected individuals, although they are often obscured by concurrent dysarthria; extracerebellar manifestations, including limbic encephalitis and peripheral neuropathy, are infrequent but have been observed [2,3]. This syndrome arises from a breakdown of immune tolerance, leading to the production of anti-Yo antibodies that target intracellular neuronal antigens. Anti-Yo PCD is associated with distinct genetic, neuropathological, and cytotoxic mechanisms linked with various malignancies, predominantly ovarian and breast cancers [1,2]. Although anti-Yo PCD predominantly affects the cerebellum (and Purkinje cells), it can also involve other brain structures, such as the brainstem and, more rarely, peripheral nerves [1].

The most common form of PCD is associated with anti-Yo antibodies, which exert cytotoxicity by binding to the 62 kDa Yo antigen, a protein located intracellularly in Purkinje cells [2,4]. This interaction occurs primarily through direct antibody binding to the Yo antigen without the involvement of immune cells such as T lymphocytes; this mechanism suggests that the antibodies may disrupt intracellular signalling and protein homeostasis in neurons, leading to cell death [4]. The findings also point to a broader trafficking mechanism of IgG antibodies within the central nervous system (CNS), where they may penetrate neuronal membranes or bind to intracellular targets, contributing to neurodegeneration [2,4]. Anti-Yo antibodies, predominantly of the IgG1 subtype, primarily target cerebellar degeneration-related protein 2 (CDR2) and its paralog, CDR2-like (CDR2L); CDR2 is involved in gene transcription regulation, while CDR2L is implicated in protein synthesis [5,6,7]. The distinct structural features of these proteins—CDR2 being a 62 kDa protein with a coiled-coil/leucine zipper domain crucial for dimerization, and CDR2L being a 55 kDa protein with three coiled-coil domains and 50% sequence identity to CDR2—underscore their functional differences [5,6,7]. CDR2 is primarily nuclear, while CDR2L is cytoplasmic, reflecting their distinct roles in the pathogenesis of PCD. Although CDR2 RNA is ubiquitously expressed, its protein is selectively found in cerebellar Purkinje neurons, some brainstem neurons, and reproductive tissues, indicating post-transcriptional regulation [5,6,7].

Pathological features of this disease include Purkinje cell axon depopulation, secondary demyelination, immune infiltrates, and microglial activation, occasionally extending beyond the cerebellum [6,8]. These antigens drive PCD by disrupting calcium homeostasis and c-Myc activity, with anti-Yo antibodies impairing CDR2-c-Myc interactions, leading to Purkinje cell apoptosis [7,9]. CDR2L overexpression and mutations further amplify immune responses, solidifying its role as the primary antigen in PCD [7,9]. Significant cerebellar cortical atrophy with near-total Purkinje cell depletion, Bergmann astrocyte proliferation, and granule cell thinning also occur. In vitro studies have demonstrated that anti-Yo antibodies induce Purkinje cell death, with early inflammation and later neuronal loss without inflammation; histopathological features include cerebellar atrophy and Purkinje cell depletion [7,9,10]. CDR2 and CDR2L are expressed in ovarian cancer tissues, with MHC-II antigen upregulation, suggesting immune dysregulation in disease progression [3,10]. Another antigen identified as a potential biomarker for anti-Yo paraneoplastic cerebellar degeneration is CDR 34, a novel 34 kDa Purkinje cell-specific protein. However, information regarding its role is still limited, particularly concerning its implications for B-cell responses [11]. The genetic alteration and overexpression of these antigens in tumour cells are believed to trigger the primary immune reaction.

In summation, anti-Yo PCD is an autoimmune disorder in which an immune response targeting Yo antigens, shared by malignancies (usually gynecological or breast) and cerebellar Purkinje cells, results in cerebellar injury. Cytotoxic CD8+ T lymphocytes infiltrate the cerebellum and mediate the initial neuronal death, recognizing Purkinje cells as targets due to the presentation of tumour-associated antigens. Anti-Yo antibodies, although highly specific markers, are not the main mediators of harm but reflect the underlying immunological dysfunction [9,10,11,12]. This process leads to apoptotic Purkinje cell death, neuroinflammation, and eventual cerebellar atrophy, appearing as progressive cerebellar ataxia and other cerebellar dysfunctions. The pathogenesis of anti-Yo PCD reveals a maladaptive immune response generated by tumour-driven antigenic mimicry. For this reason, anti-Yo PCD seems to be, primarily, cytotoxic CD8+ T-cell mediated, and the exact role of anti-Yo antibodies remains debated, as some studies suggest that they may have a minor contributory role in pathogenesis, while others view them purely as markers; the variability in disease progression and response to immunotherapy suggests that additional, less understood factors are at play [9,10,11,12]. Therefore, the objective of our review is to look closely to the potential role of the antigens CDR2 and CDR2L, as well as other relevant markers, in the pathogenesis of anti-Yo PCD to provide the reader with an updated and synthesized review considering both in vitro and in vivo studies. We will begin by discussing the molecular and immunological characteristics of CDR2 and CDR2L (and other antigens) and then discuss the pathogenesis of anti-Yo PCD.

## 2. CDR2 and CDR2L: Role and Molecular Characteristics

### 2.1. CDR2 and CRD2L: Normal Structure and Localization

CDR2 is an onconeural antigen, targeted by Yo antibodies, which has a strong association with ovarian cancer and breast cancer. CDR2 mRNA, exclusively expressed in PCD-associated tumours, was assumed to be the sole Yo antigen [13]. However, recent studies indicate that PCD patients’ sera also target CDR2L, suggesting its potential pathogenic role [13]. A supporting theory suggests that the postnatal downregulation of CDR2 in the rat cortex and low adult expression levels in human cerebellum, alongside increased CDR2L expression, position CDR2L as a likely primary immunogenic target in PCD; this differential expression pattern indicates a potentially diminished role for CDR2-specific immune responses relative to CDR2L in PCD pathogenesis [8].

The CDR2 gene is widely transcribed and expressed in immune-privileged sites, such as cerebellar Purkinje neurons, brainstem neurons, and spermatogonia; furthermore, CDR2 is present in normal ovarian tissue and tumour tissue, predominantly within vascular smooth muscle cells [10]. Raspoting et al. conducted a blot analysis of normal and cancerous human tissues (mammary, kidney, ovary, prostate, and testis) with CDR2 and CDR2L antibodies, showing single or double bands near 55 kDa and an additional band at 60 kDa across all samples [13]. Testicular and prostate cancers exhibited strong CDR2 and CDR2L expression, with increased levels compared to normal tissues; kidney tissues displayed faint bands, and controls confirmed antibody specificity [13]. When looking at the nervous system, CDR2 was expressed in several brain regions (mainly the cerebellum), with the second most prominent site of expression being the midbrain; high levels of expression were also seen in the striatum, a target of dopaminergic projections from the SN pars compacta [8].

**Figure 1:**
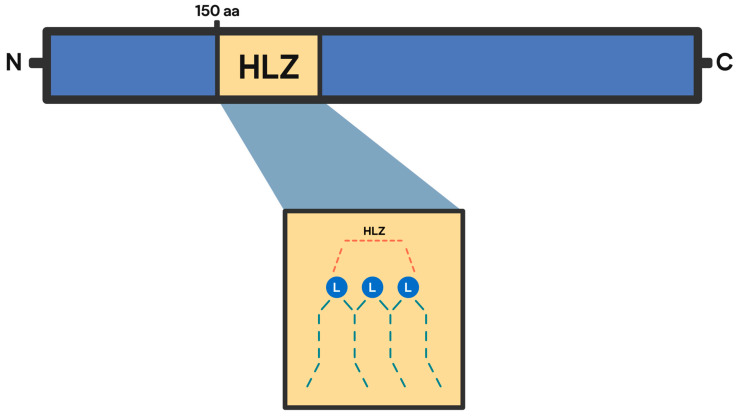
Schematic representation of the CDR2 structure, highlighting its helix–leucine zipper (HLZ) motif, located within the 150 amino-terminal regions. The HLZ motif facilitates interactions with the HLZ domain of c-Myc, mediating cellular proliferation and differentiation in various cell lines. Created by the authors (Andrea Iturralde Carrillo, Maria Montesdeoca-Lozada, 2024).

HeLa cells serve as an essential model for studying the localization and expression of CDR2 and CDR2L. Transfecting these cells allows researchers to visualize interactions with Yo antibodies, highlighting CDR2L localization at the plasma membrane and its potential pathogenic relevance [14,15]. HeLa cells have been used to evaluate the reactivity of patient sera containing CDR2 and CDR2L antibodies by transfecting the cells with the respective antigens; despite the antibodies having low avidity and giving negative results in immunohistochemistry assays, they successfully stained transfected HeLa cells, suggesting that these cells allowed for greater sensitivity in detecting low-avidity antibodies [14,15]. Furthermore, CDR2 was independently cloned from both HeLa cells and human cerebellar cDNA libraries, revealing a protein characterized by a coiled-coil/leucine zipper domain at its N-terminus [14,15]. The fluorescent immunoblotting of rat cerebellar lysate has revealed that CDR2 is a 62 kDa protein composed of 454 AA, with 150 amino-terminal regions containing a classic helix–leucine zipper (HLZ) dimerization motif (Figure 1). Its paralogue, CDR2L, has a 50% sequence identity with CDR2, a molecular mass of 55 kDa, and consists of 465 amino acids with three coiled-coil domains [9,14,15].

Kråkenes et al. studied the localization of CDR2, CDR2L, and anti-Yo antibodies in a section of the human cerebellum. They found that CDR2L was predominantly located on free ribosomes and ribosomes bound to the cytoplasm of Purkinje cells [16]. Yo antibodies consistently reacted with CDR2L in human and rat brain tissue, as well as cultured cancer cells, displaying a granular cytoplasmic staining pattern [16]. In contrast, CDR2 was primarily localized in the nuclei of neuronal cells, where Yo antibody staining was absent; in contrast, when looking at human cancer cell lines, a strong nuclear staining of CDR2 was noted, along with some cytoplasmic staining [17]. The primary gene in the tissue related to PCD is the CDR2 gene, which led to the assumption that the CDR2 protein was the primary target of anti-Yo antibodies [10,18]. However, in vivo studies of anti-Yo antibody reactivity have shown that the CDRL2 protein is the main target of these antibodies as it is intensely expressed in the tumours associated with PCD; in these tumours, anti-Yo antibodies exhibit less affinity for the CDR2 protein [17,19]. When the immune system detects the CDR2 protein, it triggers a dual response; it targets tumour cells expressing CDR2 to inhibit growth potentially, but the immune response can turn against the cerebellum, causing autoimmune cerebellar degeneration [17,19]. Both CDR2 and CDR2L are used in the detection of PCD, demonstrating equivalent sensitivity for confirmation [20]. However, CDR2L is now recognized as the major Yo antigen, as the detection of CDR2L in cell-based assays was shown to be more reliable for the diagnosis of PCD than the CDR2 antigen test which has a specificity for anti-Yo as low as 8% [21]. Testing for CDR2L in CSF reveals higher values in indirect immunofluorescence assay (IFA) and a strong reactivity in Western blot analyses [22]. Therefore, CDR2L must be included in the pathogenesis of Yo-mediated PCD [17].

Despite the prominence of CDR2L, CDR2 remains important for cerebellar cells due to its interactions with proteins involved in gene transcription and signal transduction. The presence of CDR2 is necessary for proper cellular mitosis; its absence leads to a higher incidence of aberrant mitotic spindles, resulting in the formation of daughter cells that are nonviable or genetically abnormal [23]. CDR2 interacts with proteins related to the cell cycle, including serine/threonine protein kinases activated by fatty acids and c-Myc [9]. CDR2 inhibits c-Myc-dependent transcription in tumour cell lines; CDR2 has a similar transcriptional regulatory role against NFkB, the transcription factor involved in neuronal development and synaptic plasticity, and against MRGX, the transcriptional regulator involved in cell growth, DNA repair, and apoptosis [3].

### 2.2. CDR2 and C-MYC

**Figure 2:**
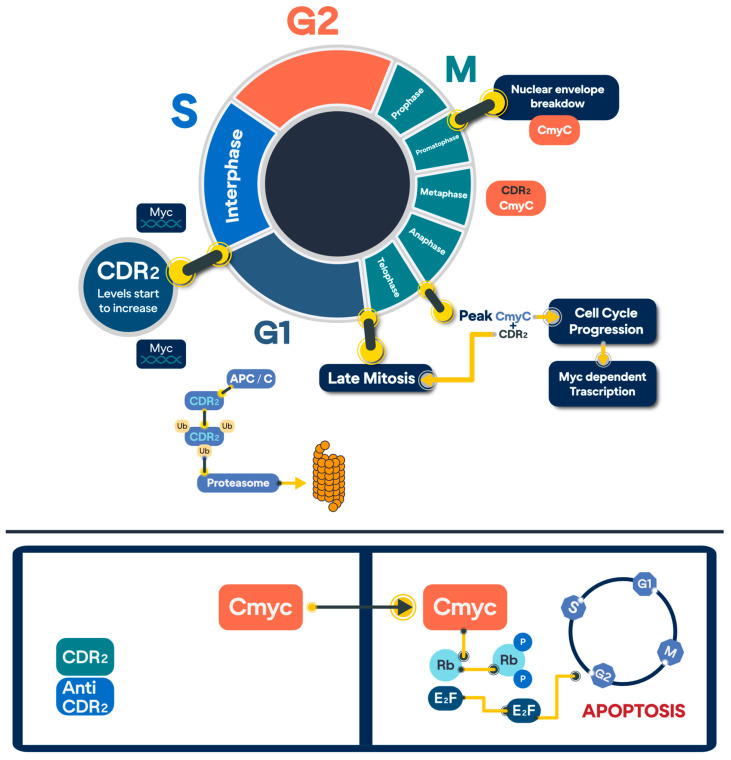
Illustration of the interaction between CDR2 and c-Myc during the cell cycle and its implications based on theories proposed by Kevin J. O’Donovan and Hirotaka J. Okano. The upper segment depicts the role of CDR2 in the cell cycle. During the G1/S phase preceding mitosis, CDR2 mRNA levels begin to increase. In prometaphase, the nuclear envelope breakdown allows CDR2 to interact with c-Myc. CDR2 levels peak during telophase, supporting continued cell cycle progression. Subsequently, CDR2 is ubiquitinated and degraded by the anaphase-promoting complex/cyclosome (APC/C). Kevin J. O’Donovan’s theory suggests that the overexpression of CDR2 leads to abnormal sequestration of c-Myc, inhibiting its activity and disrupting normal cellular processes. The lower segment represents Hirotaka J. Okano’s theory. In this model, CDR2 antibodies bind to the leucine zipper epitope of CDR2, preventing its interaction with c-Myc. As a result, c-Myc activity increases, triggering downstream pathways that lead to apoptotic cell death in Purkinje neurons. Created by the author (Andrea Iturralde Carrillo, Maria Montesdeoca-Lozada, 2024).

CDR2 is involved in gene expression and its regulation. CDR2 has a HLZ motif that interacts with the HLZ motif of c-Myc, which results in the sequestering of c-Myc in the cytoplasm; consequently, c-Myc distribution can vary between the cytoplasm and the nucleus depending on cellular proliferation and differentiation in cell lines [7]. There are two main theories regarding the interaction between CDR2 and c-Myc (Figure 2). The first, proposed by Hirotaka J. Okano et al., states that CDR2 is located perinuclearly and interacts with c-Myc, sequestering it in the cytoplasm and downregulating its activity as a nuclear transcription factor [16]. In the pathogenesis of PCD, CDR2 antibodies target the CDR2 leucine zipper epitope, sequestering CDR2 and preventing its interaction with c-Myc; this increases c-Myc activity, activating different pathways, which induces apoptosis in Purkinje neurons [16]. The second theory, proposed by Kevin J. O’Donovan et al., states that CDR2 is required for the proper execution of mitosis [7]. During the prometaphase of mitosis, the nuclear envelope breaks down, allowing CDR2 to access c-Myc; in metaphase, CDR2 starts to interact with c-Myc near the spindle poles, and CDR2’s concentration peaks during telophase, after which it is ubiquitinated and degraded by the anaphase-promoting complex/cyclosome (APC/C) [7]. In tumoral pathogenesis, the overexpression of CDR2 causes aberrant sequestration of c-Myc, inhibiting its activity, disrupting mitotic exit, and driving tumour proliferation [7].

### 2.3. CDR2 and Calcium Homeostasis

Calcium homeostasis is closely regulated in Purkinje cells due to its critical role in maintaining cellular function and integrity; disruptions in free intracellular calcium concentrations can lead to neuronal death [24]. Therefore, multiple proteins interact to maintain mitochondrial and cytoplasmatic calcium levels. For instance, calbindin D28k is an endogenous calcium buffer protein in Purkinje cells and has been linked to neurodegeneration in PCD when depleted [25]. Calbindin may help stabilize CDR2 expression and function. It has been shown that anti-Yo antibodies bind to CDR2 and CDR2L, leading to changes in intracellular calcium homeostasis, partly due to calbindin malfunction; calcium-sensitive mitochondrial-associated signalling can also be affected [9]. This suggests an essential role of calcium in neuronal degeneration and death. Furthermore, CDR2 found in the cerebellum, specifically in the neuronal cytoplasm and the proximal dendrites of the Purkinje neurons, interacts with c-myc to modulate calcium expression and calbindin buffering [26]. Meanwhile, CDR2L is linked to plasma membrane signalling involved in voltage-gated calcium channels (VGCC) and AMPA receptor-mediated calcium flux regulation [26]. Panja et al. demonstrated that anti-Yo internalization disrupts the calcium-dependant VGCC–PKC—calbindin signalling pathway and calpain-2-over-activation; it affects the complex I of the respiratory chain, increasing the production of reactive oxygen species, reducing mitochondrial membrane potential, and impairing its respiration [26]. It also affects the calcium (inward) and anion (outward) transport of mitochondrial and plasma membranes that act as defence mechanisms to counteract calcium overload [26]. The disruption of calcium homeostasis induced by CDR2 and CDR2L antibodies has been established, although their underlying mechanisms and potential therapeutic applications require further research.

### 2.4. CDR2 and CDR2L Genetic Alterations

Recent data suggest that genetic mutations in the CDR2 and CDR2L genes are key triggers for the breakdown of immune tolerance in the development of Yo-PCD. Mutations in these genes lead to the production of neoepitopes—novel antigenic peptides generated by genetic alterations in tumour cells—which are recognized by specific T-cell effectors [9,27]. These T cells cross-recognize unmutated epitopes on cerebellar Purkinje cells, triggering an autoimmune response through neo-antigenicity [9,27]. However, the generation of neoantigens alone does not fully explain the immune tolerance breakdown. The gain in copy number, resulting in onconeural antigen overexpression, may also serve as an independent or additional trigger for immune system activation [12]. The amplification of CDR2L (≥6 copies) was primarily observed, leading to increased CDR2L transcription and protein expression in Yo-PCD breast cancers (BCs), along with upregulated immune response pathways and dominant IgG-plasma cell infiltration; these tumours, associated with Yo-PCD, exhibit distinct morpho-phenotypic and biomolecular profiles, characterized by invasive HER2-driven carcinoma with mutated CDR2L overexpression and early metastasis to regional lymph nodes, despite heavy immune cell infiltration [12].

In a study by Small et al., 65% of Yo-PCD ovarian tumours were found to harbour at least one somatic mutation in Yo antigen genes, which were predominantly missense mutations. Additionally, recurrent gains in the CDR2L gene were observed in 59% of Yo-PCD patients, leading to tumour protein overexpression. They also observed that CDR2L is overexpressed in most Yo-PCD ovarian carcinomas, regardless of gene amplification, suggesting that alternative mechanisms drive protein upregulation [27]. This overexpression likely plays a central role in initiating the autoimmune response, as some tumours with amplification lacked mutations. While the mechanisms behind immune tolerance breakdown may vary, all Yo-PCD patients exhibited either Yo gene amplification/protein overexpression or mutation, suggesting that these alterations are sufficient to trigger Yo disorders [27].

Peters et al. studied anti-Yo PCD associated with breast cancer and found antigen amplification in twelve of the seventeen tumours, with CDR2L amplification in nine of the fourteen samples (64.3%) and CDR2 amplification in two of the fourteen samples (14.3%) [12]. RNA sequencing confirmed no differential expression of CDR2 but showed a significant overexpression of CDR2L transcripts in Yo-PCD breast cancer compared with controls. These findings support the hypothesis that CDR2L is the major antigen in Yo-PCD [12]. Furthermore, due to the co-localization of ERBB2 and CDR2L on chromosome 17, a potential association between ERBB2 gene amplification and HER2 overexpression with CDR2L overexpression is suggested, though this mechanism remains unclear [12]. These genetic alterations, implicated in ovarian and breast cancers, may represent a broader biomolecular signature of Yo-PCD, emphasizing the need for further exploration to inform diagnostic and therapeutic strategies for the disorder.

### 2.5. CDR 34 as an Antigen in Anti-Yo PCD

CDR 34, a 34 kDa Purkinje cell-specific antigen, was identified through cerebellar cDNA and confirmed as a potential biomarker for PCD [11,28]. Detecting the 34 kDa Purkinje cell antigen (CDR 34) in tumour tissue from PCD patients using human serum proved challenging due to nonspecific bands of similar size; the CDR 34 sequence contains 91% tandem repeats of a six-amino-acid motif, identified as a B-cell epitope, indicating its role in antibody responses [11]. However, Western blot analysis with a purified rabbit anti-34 kDa antibody successfully identified CDR 34 exclusively in PCD tumour tissues, with no expression detected in normal tissues or tumours from non-PCD patients [11,28]. CDR 34 protein is specifically expressed in Purkinje cells, with a 500-fold mRNA enrichment in these cells within the cerebellum; although CDR 34 mRNA shows minimal expression in non-cerebellar tissues, current immunohistochemistry techniques lack the sensitivity to detect such low levels. Furthermore, in situ hybridization is needed to explore its potential expression in select cortical neurons [11,28].

## 3. General Characteristics and Pathogenesis of Anti-Yo PCD

### 3.1. Epidemiology and Clinical Characteristics

Anti-Yo PCD associated with gynecological cancer is distinguished by rapidly progressive cerebellar ataxia, which accounts for approximately 50% of all PCD cases [3]. Patients typically have a diagnosis of cancer at the time of presenting with cerebellar ataxia, with ovarian and breast cancers being the most frequently observed [29,30]. In 1919, Brouwer first described a case of a 60-year-old woman with subacute cerebellar syndrome associated with polymorphic sarcoma of the pelvis [31]. In 1983, Greenlee and Brashear first identified anti-Purkinje cell antibodies in two ovarian carcinoma patients with PCD, a finding later confirmed by Jaeckle et al. in 1985 in six of twelve patients; this immunological origin of the syndrome was further validated and expanded by Darnell and Posner in 2003 [32,33]. This discovery led to the theory that immune-mediated pathophysiological mechanisms affect the cerebellum in the presence of cancer [31].

Anti-Yo PCD is the second most common immune-mediated cerebellar ataxias associated with a high-risk phenotype [29,31]. Vogrig et al. reported an annual incidence of 0.89/100,000 people for paraneoplastic neurological syndromes (PNS), with anti-Yo PCD accounting for 30% of cases [34]. Few case series report on the prevalence of anti-Yo PCD; one notable study found a prevalence of 2.3% (13/557 cases) among patients with ovarian cancer and 1.6% (4/253 cases) among those with breast cancer [3]. Despite the increasing interest in anti-Yo PCD, comprehensive population data remain limited. However, identifying autoimmune characteristics and the presence of specific antigens and antibodies have contributed to an increase in diagnosis rate.

Patients with PCD typically present with rapidly progressive cerebellar symptoms, often preceded by flu-like prodromes. Gait ataxia, usually asymmetric, is the initial and most prominent manifestation. As the condition progresses, pan-cerebellar dysfunction affects the trunk and upper extremities [29,31]. Isolated cerebellar symptoms are more frequent in anti-Yo PCD compared to other types of PCD, occurring in about 40% of patients [29,31]. Due to the wide variety of symptoms and signs in anti-Yo PCD, there are no unified specific diagnostic criteria. In 2021, experts classified anti-Yo PCD as a rapidly progressive cerebellar syndrome with a high-risk phenotype due to its >90% cancer association and 80% antibody detection rate; when cancer is present, it is categorized as a definitive paraneoplastic neurologic syndrome (PNS), based on clinical and antibody criteria [29,35].

In anti-Yo PCD, cerebrospinal fluid (CSF) abnormalities, including mild pleocytosis, elevated protein levels, and oligoclonal bands, are observed in 93% of cases; these abnormalities tend to normalize within three months, indicating a transient inflammatory response [3]. Following the phenotype and serological confirmation of anti-Yo PCD, screening for an underlying malignancy is essential as PCD often precedes or coincides with early tumour stages. A multidisciplinary approach is advised, with biannual screenings if initial tests are negative, as 90% of associated tumours are identified within one year; if the initial screening does not reveal cancer, periodic screening every six months should be undertaken [36]. The low prevalence of anti-Yo PCD has hindered randomized controlled trials, leading to a lack of evidence-based treatment guidelines [2]. Immunotherapies, including steroids, plasmapheresis, and IVIG, have shown limited efficacy; tumour treatment is the most effective approach, with some cases showing partial improvement following tumour-specific therapies and immunosuppressive treatments, but overall response rates remain low [3].

### 3.2. Neuropathology of Anti-Yo PCD

While the in vivo role of anti-Yo antibodies in cerebellar injury remains unverified, in vitro studies demonstrate that they induce Purkinje cell death independent of T lymphocytes [3]. Early PCD pathology includes mild perivascular lymphocyte cuffing, microglial activation, and CD8 lymphocyte infiltration within the cerebellar Purkinje cell layer [3,4,5]. Inflammatory infiltrates may also be present in the brainstem and cerebral cortex, though they occur at a lower magnitude than in the cerebellum. As the disease progresses, rapid Purkinje cell loss occurs without inflammation, suggesting a “burn-out” phase due to reduced immune response and extensive neuronal loss [3,4,5].

Greenlee et al. found that microglia and macrophages accumulated near Purkinje cells only after cell death; in cultures treated with anti-Yo antiserum, CD11b-positive cells were absent at 48 and 72 h despite extensive cell death [37]. As cell death progressed, macrophage and microglial infiltrates became increasingly abundant, suggesting that brain mononuclear cells were unlikely to be the initial cause of Purkinje cell damage. The macroscopic finding included significant cerebellar cortical atrophy with near-total Purkinje cell depletion and Bergmann astrocyte proliferation, showing granule cell thinning [37]. Scattered T-cell lymphocytes were found in the leptomeninges and around blood vessels in the dentate nucleus, but no inflammatory infiltrates were present in the cerebellar cortex [37].

Among the histopathological findings, the use of immunohistochemistry in the study of anti-Yo PCD should be highlighted. Pathologic findings in ovarian cancer tissues showed CDR2 and CDR2L proteins expressed with variable staining patterns, primarily cytoplasmic, and nuclear staining for CDR2 in mitotic cells, while Purkinje cells showed a patchy cytoplasmatic pattern [13]. Western blot analysis identified bands around 55–60 kDa, indicating their presence in cancer cells, with some expression also seen in normal ovarian tissues. Increased expression of MHC-II antigens was present particularly around vessels in the Purkinje cell layer. The immune response to these proteins likely involves multiple immunological host factors, including the breakdown of immune tolerance [13].

### 3.3. Autoimmune Pathophysiological Mechanisms

Anti-Yo PCD extensively destroys Purkinje cells; the expression of CDR2/CDR2L in both Purkinje cells and neoplastic cells suggests a cross-reactivation of the immune system [6]. However, different types of cancers, including ovarian, mammary, testicular, and prostate cancer, show increased expression of CDR2 and CDR2L without association to PCD development [13]. Autoimmunity against Purkinje cells involve other factors other than the expression of CDR2/CDR2L in tumour cells. Hillary et al. demonstrated a significant genetic association between certain HLA haplotypes and anti-Yo PCD in patients with gynecological cancers. The study identified protective HLA haplotypes, notably DPA101:03~DPB104:01 and DRB104:01~DQA103:03; conversely, the haplotype DRB113:01~DQA101:03~DQB1*06:03 was associated with increased risk in ovarian cancer cases, while the DRB1*04:01 association was the most robust, and its imputation accuracy was only about 80%, indicating a need for further subtype analysis [38]. The expression of CDR2 and CDR2L may interact with HLA pathways, potentially influencing immune responses and susceptibility to autoimmune conditions like PCD [38]. These results suggest that multiple epitopes within Yo proteins or interactions with additional unknown antigens may be involved in the pathogenesis of anti-Yo PCD. The hypothesis of a direct attack on Purkinje cells by reactive lymphocytes in PCD has not been proven. The involvement of T lymphocytes has been documented in different studies. Immunohistochemical analysis has shown CD8 lymphocyte infiltration and diffuse microglial activation [37]. A study looking at cerebellar samples from two patients with PCD showed microglial nodules within the cortical Purkinje cells layer and cytotoxic CD8 granzyme B-positive T-cell infiltrate [39]. Tanaka et al. demonstrated that cytotoxic T-cell (CTL) activity against the Yo peptide was present in a case series of patients with HLA A24 [39]. Furthermore, Yshii et al., created a mouse model where the blockade of CTLA4, a protein receptor on T cells that acts as an immune checkpoint, produced paraneoplastic neurologic disorders [40]. In this model, they observed a T cell-driven IFN-γ receptor/pSTAT1 signalling pathway in Purkinje neurons. They later obtained complete protection of Purkinje cells from cytotoxic T cells by neutralizing IFN-γ [40].

The breakdown of immune tolerance is necessary for PCD to develop. CDR2/CDR2L proteins have been found in thymic medullary epithelial cells, suggesting a central immune tolerance process to these proteins [6]. In anti-Yo PCD, specific IgGs bind to intracellular epitopes of Purkinje cells, initiating the process of depopulation and immune infiltrate within the cerebellum, which indicates the pathologic recognition of autoantibodies. This breakdown may be due to an excessive presentation of normal and mutated CDR2/CDR2L proteins in tumour cells, leading to the pathological recognition of these antigens and the production of antibodies [19,27]. The exact mechanism, however, has not been completely elucidated. Yshii et al. reported that inflammatory processes that result in an upregulation of MHC class I molecules in Purkinje cells could facilitate CD8 T-cell recognition of antigens in the cerebellum [6]. The later migration of cytotoxic CD8 T cells to the CNS would require the disruption of the brain blood barrier (BBB) in the cerebellum. Adhesion molecules expressed in the endothelium help cell migration through the BBB and are upregulated during inflammatory processes. Specifically, CD8 T-cell migration is facilitated by α4β1-integrin, VCAM-1, and JAM-B proteins [41,42]. The activation of CD8 T cells releases granzyme B- and perforin-containing cytolytic granules [43]. This results in an extensive loss of Purkinje cells associated with inflammatory infiltrate presenting on CD8 cells, macrophages, and activated microglia [38].

Finally, Greenlee et al. proposed a different model of Purkinje cell death secondary to the intracellular interaction of anti-Yo with CDR2 and CDR2L that disrupts cellular homeostasis. This is based on the evidence of normal immunoglobulins adsorption by Purkinje cells shown in a rat model by Hill et al. [44]. Under normal circumstances, the immunoglobulins would be cleared from the intracellular fluid, but in patients with PCD, anti-Yo IgGs bind to a 62 kDa cytoplasmic protein, altering the functional processes of Purkinje cells, such as calcium regulation, causing subsequent cell death [26]. In his model, Greenlee et al. showed that macrophage and microglial responses were secondary to the cell death process and were not a trigger of Purkinje cell death. They concluded that Purkinje cell death in cultures treated with anti-Yo IgG is due to the specific interaction between anti-Yo IgG and the 62 kDa Yo antigen and not merely from general intracellular antibody uptake and accumulation [2].

### 3.4. Future Directions

Research in this field is continually evolving, with future focus mainly on the molecular and genetic bases of the disease, as well as on the immune response that triggers Purkinje cell death. With further research and advancements in comprehension of the disease, diagnostic and treatment approaches should incorporate novel diagnostic techniques and therapies, such as immunological checkpoint inhibitors or genetic approaches targeting miRNA.

Yshii et al. have proven that the inhibition of IFNγ with a monoclonal antibody since day 0 of exposure to anti-Yo antibodies can prevent the development of PCD in a mouse model, showcasing that IFNγ is a key chemokine in the inflammatory cascade of Purkinje cells exposed to anti-Yo antibodies [6]. Using an IFN-γ–neutralizing antibody prevented destruction of the neurons and limited T-cell infiltration of the cerebellum [6,19,40]; however, further research is needed to prove its safety and efficacy in humans. The availability of Emapalumab, a full-human monoclonal anti–IFN-γ antibody, presents a promising option for further research in the treatment of this disease [6,19,40].

Tveit Solheim et al. found that exosomal miRNA profiles differ between patients with ovary cancer and anti-Yo PCD compared to patients with ovary cancer alone, suggesting the possibility of using exosomal miRNAs as biomarkers for PCD and as contributors to the pathogenesis of the disease [45]. Patients with PCD and ovary cancer express several miRNAs that could aid in diagnosis; for instance, miR-15b-5p, miR-20b-5p, miR-21-5p, miR-34a-5p, miR-146a-5p, and miR-340-5 are dysregulated in PCD [45]. Notably, miR-146a-5p, which acts as an anti-inflammatory mediator, is downregulated, while miR-34a, which acts as a regulator of T-cell activation, is upregulated in PCD [45]. Furthermore, miRNAs also influence the interaction between CDR2L and the ribosomal protein RPS6; miR-486-5p, miR-98-5p, miR-25-3p, miR-20b-5p, and miR-16-5p, which are expressed in PCD patients, target the CDR2L and RPS6 genes, as identified through the DIANA-TarBase database of experimentally validated miRNA–gene interactions [45]. The aforementioned evidence showcases that exosomal miRNA dysregulation may contribute to the immunopathology seen in paraneoplastic neurological syndromes and could be used as a potential biomarker for diagnosis.

## 4. Conclusions

Anti-Yo PCD is a cerebellar disorder characterized by a high-risk phenotype and the presence of antibodies targeting CDR2/CDR2L. It is the second most common cause of immune-mediated cerebellar ataxias and the leading cause of isolated cerebellar dysfunction. The disorder is marked by the presence of anti-Yo antibodies, which attack the onconeural antigens CDR2 and CDR2L, primarily located in the cytoplasm of Purkinje cells. PCD highlights the aberrant expression of these normally immune-privileged antigens in tumours, potentially triggering abnormal cell growth and programmed cell death pathways. The immune response causes early lymphocytic infiltration, followed by Purkinje cell loss without inflammation in the later stages. Anti-Yo antibodies induce cell death via CDR2 interaction, underscoring the role of immune dysregulation in PCD progression. In vitro studies demonstrate that anti-Yo antibodies induce Purkinje cell death via CDR2 binding, independent of immune cell involvement. Disease progression is marked by neuronal loss and immune “burn-out”, suggesting a breakdown in immune tolerance. Additionally, CD8+ cytotoxic T cells play a pivotal role in targeting these epitopes within Purkinje cells, leading to cell death. The influence of specific HLA haplotypes on susceptibility further complicates the interplay between immune responses and tumour-associated antigen presentation. The pathogenesis reveals a maladaptive immune response generated by tumour-driven antigenic mimicry. For that reason, anti-Yo PCD seems to be, primarily, cytotoxic CD8+ T-cell mediated, and the exact role of anti-Yo antibodies remains debated, as some studies suggest that they may have a minor contributory role in pathogenesis, while others view them purely as markers; the variability in disease progression and response to immunotherapy suggests additional, less understood factors at play. Continued investigation into these immune mechanisms is essential for elucidating the underlying pathways of anti-Yo PCD.

The molecular characterization of CDR2 and CDR2L emphasizes their critical roles in PCD pathogenesis. CDR2 regulates mitosis and transcription through interactions with signalling molecules such as c-Myc, NFkB, and MRGX. CDR2L, which shares 50% sequence identity with CDR2, is overexpressed in tumour tissues and is the primary target of anti-Yo antibodies, offering higher diagnostic sensitivity for PCD. These antibodies disrupt calcium homeostasis in Purkinje cells, impairing calbindin signalling and mitochondrial function, leading to neurodegeneration. Genetic alterations in CDR2 and CDR2L, including mutations and amplifications, trigger autoimmune responses, highlighting CDR2L as a key antigen in Yo-PCD pathogenesis. The early detection of PCD can facilitate the identification of associated cancers, underscoring the need for further research into the properties of CDR2/CDR2L and the mechanisms behind the antibodies targeting them. Understanding these elements is crucial for developing improved diagnostic and prognostic tools for PCD and its associated malignancies.

## Data Availability

The original contributions presented in the study are included in the article, further inquiries can be directed to the corresponding authors.
